# Developing a person-centered stated preference survey for dementia with Lewy bodies: value of a personal and public involvement process

**DOI:** 10.3389/frdem.2024.1421556

**Published:** 2024-07-15

**Authors:** Paula Sinead Donnelly, Aoife Sweeney, Emily Wilson, Anthony Peter Passmore, Noleen K. McCorry, Marco Boeri, Joseph P. M. Kane

**Affiliations:** ^1^Centre for Public Health, School of Medicine, Dentistry and Biomedical Sciences, Queen's University Belfast, Belfast, United Kingdom; ^2^Northern Ireland Lewy Body Dementia Research Advisory Group, Queen's University Belfast, Belfast, United Kingdom; ^3^Patient-Centered Outcomes, OPEN Health, London, United Kingdom

**Keywords:** patient and public involvement, dementia with Lewy bodies, stated preference survey, discrete choice experiment, best-worst scaling, patient perspective, preferences, pretesting

## Abstract

**Introduction:**

The development of high-quality stated preference (SP) surveys requires a rigorous design process involving engagement with representatives from the target population. However, while transparency in the reporting of the development of SP surveys is encouraged, few studies report on this process and the outcomes. Recommended stages of instrument development includes both steps for stakeholder/end-user engagement and pretesting. Pretesting typically involves interviews, often across multiple waves, with improvements made at each wave; pretesting is therefore resource intensive. The aims of this paper are to report on the outcomes of collaboration with a Lewy body dementia research advisory group during the design phase of a SP survey. We also evaluate an alternative approach to instrument development, necessitated by a resource constrained context.

**Method:**

The approach involved conducting the stages of end-user engagement and pretesting together during a public involvement event. A hybrid approach involving a focus group with breakout interviews was employed. Feedback from contributors informed the evolution of the survey instrument.

**Results:**

Changes to the survey instrument were organized into four categories: attribute modifications; choice task presentation and understanding; information presentation, clarity and content; and best-best scaling presentation. The hybrid approach facilitated group brainstorming while still allowing the researcher to assess the feasibility of choice tasks in an interview setting. However, greater individual exploration and the opportunity to trial iterative improvements across waves was not feasible with this approach.

**Discussion:**

Involvement of the research advisory group resulted in a more person-centered survey design. In a context constrained by time and budget, and with consideration of the capacity and vulnerability of the target population, the approach taken was a feasible and pragmatic mechanism for improving the design of a SP survey.

## 1 Introduction

Lewy body dementia (LBD), encompassing both dementia with Lewy bodies (DLB) and Parkinson's disease dementia (PDD), is recognized as the second most common dementia subtype (Vann Jones and O'Brien, 2014; McKeith et al., 2017; Kane et al., 2018). DLB is characterized by four “core” clinical features/symptoms: fluctuating cognition, recurrent visual hallucinations, REM sleep behavior disorder, and spontaneous parkinsonism (McKeith et al., 2017). However, additional symptoms may include severe neuroleptic sensitivity, postural instability, repeated falls, syncope, severe autonomic dysfunction, hypersomnia, hyposmia, hallucinations in other modalities, systematized delusions, apathy, anxiety and depression. There is no staging system for DLB and experiences are diverse, however the disease course is invariably progressive (Matar et al., 2021) and associated with a poorer prognosis than for other forms of dementia (Mueller et al., 2017).

Understanding patient preferences is critical for pursuing meaningful and relevant avenues of research. Health preference research aims to understand the values and preferences of key stakeholders to inform person-centered care, research and policy. Within this realm, stated preference (SP) methods have emerged as a means of quantifying patient preference information (Soekhai et al., 2019b). Two well-established SP methods in healthcare research are discrete choice experiments (DCEs) and best-worst scaling (BWS) (Soekhai et al., 2019a; Hollin et al., 2022). DCEs present respondents with a series of hypothetical alternatives (e.g., hypothetical treatment A or B) and ask them to select their preferred option, aiming to elicit preferences, explore the relative importance of attributes (e.g., cost, efficacy, and risk), and understand which tradeoffs respondents are willing to accept between the benefits and risks of adverse events or cost (e.g., a willingness to accept a higher risk of side effects for greater treatment efficacy). Each DCE choice task typically includes two or three alternatives, which might or might not also contain an opt-out alternative (e.g., choosing no treatment) or the standard of care (e.g., treatment as usual). On the other hand, BWS requires respondents to identify the “best” and “worst” items from a set of items (for example, side-effects, mode of administration and frequency of administration). Each BWS choice task typically includes three, five or seven items. Elicited preferences are contingent on how the scenario and the attributes (or items) are described. Ensuring the appropriate specification of the attributes is therefore essential for designing a valid instrument and collecting reliable preference data.

The recommended steps in the instrument development process of SP surveys are evidence synthesis, expert input, end-user engagement, pretest interviews and pilot testing (Janssen et al., 2016; Campoamor et al., 2024). The aim of end-user engagement is to improve an instrument's person-centeredness. This may involve establishing an advisory board, comprising key stakeholders, who are actively involved throughout the study (Janssen et al., 2016). This step is reflective of the shifting paradigm toward person-centered research as well as personal and public involvement (PPI) in research.

There is a clear theoretical framework supporting PPI in healthcare research (Rose, 2014; Frith, 2023). In dementia research, the Alzheimer's Society Research Network, formerly known as the Quality Research in Dementia (QRD) network, was founded on the principle that individuals with dementia and care partners can provide unique and valuable contributions to research (Alzheimer's Society, 2019). This network has served as a beacon for PPI in dementia research. Work associated with the Edinburgh Centre for Research on the Experience of Dementia (ECRED) has also exemplified the value of including a research advisory group (RAG) early in a study (e.g., Watchman et al., 2024). Guidelines and resources have been established to assist researchers in effectively integrating meaningful PPI in their research (Crowe et al., 2020; UK Research and Innovation, 2024). There is also increasing recognition of the potential for preference research to benefit from PPI (Aguiar et al., 2021; Shields et al., 2021). However, despite the importance of PPI in health economics and preference research, there is no guidance on establishing effective PPI in preference studies.

Pretesting is a flexible process where representatives from the target population are engaged in improving the validity, reliability, and relevance of the survey (Campoamor et al., 2024). This is achieved by, for example, refining the survey's content and structure, reducing sources of unnecessary burden and advising on potential ethical issues. Pretest interviews involve presenting the survey instrument to people similar to the final respondents, asking them to respond to the survey thinking out loud. The survey instrument is then updated based on feedback. The International Society for Pharmacoeconomics and Outcomes Research (ISPOR) Task Force therefore recommends pretesting as part of a rigorous design process (Bridges et al., 2011). However, despite the importance of pretesting and calls for transparency in the survey development process, there are few studies detailing the process and outcomes (Vass et al., 2017; Pearce et al., 2021).

Both qualitative and quantitative methods have been recommended in pretesting (Johnston et al., 2017; Vass et al., 2017; Hollin et al., 2020). Cognitive interviewing utilizing a verbal protocol analytical technique called “*think aloud*” is one pretesting approach, while focus groups and observations of participants silently completing survey tasks represent other approaches (Mariel et al., 2021; Pearce et al., 2021; Haggar et al., 2022; Campoamor et al., 2024). Co-design approaches, wherein respondents actively participate to solve issues together with the research team, may also be utilized (Aguiar et al., 2021; Campoamor et al., 2024). In this regard, pretesting is a collaborative process and has been aptly described as a “codevelopment type of engagement” (Campoamor et al., 2024).

The necessary extent of pretesting is case-specific (Mariel et al., 2021); however, pretesting typically occurs across multiple waves of survey administration, with improvements iteratively incorporated at each wave. For DCEs in environmental valuation (i.e., DCEs exploring environmental resources), it has been suggested that around two to eight focus groups, five to ten cognitive interviews, and one to two pilot surveys is sufficient (Mariel et al., 2021). Traditional approaches to pretesting are therefore resource intensive in terms of time, recruitment and costs associated with remunerating participants for their time. Participants may also experience significant demands on their time and potential burden. Furthermore, whereas in traditional pretesting different members of the target population are involved at each wave, DLB is a hard-to-reach population which has led to challenges with research participation (Goldman et al., 2020).

In a reflexive essay, drawing on insights from academic researchers at the ECRED, as well as firsthand experiences of a person living with dementia actively involved in research and a facilitator of the ECREDibles- a group of people living with dementia who share an interest in research- the authors emphasize the critical importance of prioritizing the wellbeing of individuals with dementia in research endeavors (Warran et al., 2023). This highlights the necessity of balancing the importance of pretesting with the potential burden traditional approaches may impose on a vulnerable population of individuals with DLB. Consequently, we opted for an alternative approach to the instrument development process.

In this study, the alternative approach to the instrument development process involved conducting the stages of end-user engagement and pretesting simultaneously with a PPI RAG. A co-design approach was adopted with RAG contributors actively encouraged to provide input to refine the survey. The aims of this paper are to (1) report on the outcomes of collaboration with the PPI RAG during the design phase of a SP survey incorporating a DCE and best-best scaling [BBS; a variation of BWS (Huls et al., 2022)], that measured treatment preferences of individuals with DLB and their care partners, and (2) evaluate the strengths and limitations of this alternative approach to instrument development as a pragmatic alternative to traditional design approaches for SP surveys. This was a unique circumstance given that DCEs and BBS have not yet been used with this population, and consideration of the potential burden that extensive pretesting approaches may impose on this vulnerable population was required.

## 2 Materials and methods

### 2.1 Personal and public involvement

To facilitate the development of a SP survey instrument for individuals with DLB and their care partners, input from the Northern Ireland Lewy Body Dementia Research Advisory Group (LBD RAG) was sought during the design phase of the survey. The LBD RAG, comprising individuals with LBD and their care partners, assessed the patient-centeredness, acceptability and accessibility of the survey instrument and provided advice on ethical considerations.

The LBD RAG contributors were recruited through advertisements in local Psychiatry of Old Age services, including a LBD clinic, and social media networks. There were no exclusion criteria applied for membership in the RAG in order to capture a wide range of perspectives and experiences. Interested individuals provided contact information to their clinician, who was a member of the research team (JK). Subsequently, the study coordinator (PSD) contacted potential contributors to explain the PPI initiative and the role of the RAG within the study. Ten RAG contributors, comprising four individuals diagnosed with LBD and six care partners, one of whom is a co-author (EW), attended the involvement event.

Guidelines set forth by the National Institute for Health Research (NIHR) were followed regarding the remuneration of individuals' time and reimbursement of expenses (NIHR, 2023). The Guidance for Reporting Involvement of Patients and the Public 2 Short Form (GRIPP2-SF) (Staniszewska et al., 2017) was used to summarize PPI involvement in the current study (Table 1).

**Table 1:** Patient (personal) and public involvement in the development of a stated preference survey reported using GRIPP2-SF.

**Section and topic**	**Item**
1. Aim/s	The aim of personal and public Involvement (PPI) in the study was to co-design a person-centered stated preference (SP) survey that was acceptable to, and accessible for, people with dementia with Lewy bodies (DLB) and their care partners.
2. Methods	Involvement of the Northern Ireland Lewy Body Dementia Research Advisory Group (LBD RAG), which comprises individuals with LBD and their care partners, took place as a half-day event in September 2023. RAG contributors were recruited through advertisements in local Psychiatry of Old Age services, including a LBD clinic, and social media networks. Ten RAG contributors, comprising four individuals diagnosed with LBD and six care partners, attended the involvement event. A focus group with breakout interviews was carried out. The focus group involved improving the content and clarity of key study documentation. The interviews involved RAG contributors completing example choice tasks and providing feedback on how the accessibility of the tasks could be improved for potential participants. Modifications arising from RAG recommendations were categorized *post-hoc*. Feedback from the contributors on their experience of involvement was collected informally through phone calls conducted by the study coordinator (PSD). Guidelines set forth by the National Institute for Health Research (NIHR) were adhered to regarding the remuneration of individuals' time and reimbursement of expenses. One care partner from the RAG is a co-author on this paper (EW) having made valuable contributions to paper edits.
3. Results	RAG contributors contributed significantly to the evolution of the survey instrument. The ways in which contributors informed the study included: Providing recommendations aimed at enhancing the clarity of the discrete choice experiment (DCE) attribute descriptions. • Suggesting improvements to make the presentation of the DCE and best-best scaling (BBS) choice tasks more user-friendly. • Sharing their preferences regarding the presentation of information. • Offering advice on enhancing the survey's clarity and usability. • Providing suggestions for new questions or items that are relevant to the research question. • Advising on mitigating potential ethical issues. • Recommending ways to reduce sources of unnecessary burden within the survey. • Suggesting suitable ways to explain the study to potential participants.
4. Discussion	Involvement of the RAG at this stage of the study was very effective and influenced the evolution of the survey instrument, based on the impacts in Section 3. The RAG suggested changes to and highlighted issues within the survey, leading to a more person-centered SP survey that better met the needs of people with DLB. Given that the reliability of findings from preference studies is reliant on the quality of the choice methods, partnership with the RAG will ultimately lead to more accurate preference findings. Improving the acceptability and accessibility of the design will also ultimately benefit recruitment to the study. RAG contributors were informed about the research methods used in the study which likely positively contributed to the quality of the feedback they provided. In addition, possible power imbalances were managed by offering contributors reimbursement for their expenses and remuneration for their time. At the outset of the involvement event, the research team emphasized that the nature of the partnership would be shaped through communication between the researchers and lay members. This helped to create a positive environment which may also have ensured contributors felt confident and supported in sharing their views. However, there were limitations. Challenges with conducting PPI in the design of preference studies has been acknowledged, including the need for adequate training in preference research methods (Goodwin et al., 2018; Al-Janabi et al., 2021). In the current study, RAG contributors were introduced to the concept of DCEs and BBS, but they were not provided with structured training on these methods. It is therefore possible that this limited the input from contributors. Additionally, some care partners reported reluctance to express or elaborate on their opinions in the presence of the individual with DLB. Future studies may consider utilizing individual interviews or focus groups to overcome this.
5. Reflections	The RAG partnership played a critical role in informing the design of the SP survey. Partnership was sought during the end-user engagement and pretesting phase of the study, but ideally, RAG contributors would have contributed toward the design of the DCE attributes or earlier in the formulation of the research question. However, due to the extensive range of possible DLB symptoms and the resulting complexity it would impose on the DCE design if all possible symptoms were included as attributes, this approach was deemed impractical. Consequently, advisory input was sought at a subsequent stage, and the DCE design was informed by clinical experts with a focus on the key diagnostic symptoms of DLB. Nonetheless, the RAG contributors in this study contributed to the evolution of the study design and influenced its progression, thereby contributing meaningfully to the study. We will continue to collaborate with the LBD RAG throughout the research study. Although all RAG contributors shared positive reflections on the involvement process, some contributors reported feeling fatigued during the event. We are aware that this might have limited the extent to which some contributors were able to engage. Some individuals with more advanced cognitive impairment may have also found it more challenging to contribute fully to the focus group discussions. However, we felt that the breakout interviews helped to ensure that those wanting to share their views had an opportunity to do so. This therefore facilitated more inclusive opportunities for people at different stages of DLB to express their views and contribute to the survey evolution.

### 2.2 The draft survey instrument

A draft survey instrument was developed to address the research question, “Which symptoms would individuals with DLB and their care partners most like to see improved upon by a potential therapy?” Specifically, the survey instrument was designed to assess the relative importance of DLB symptoms regarding priorities for treatment, how individuals trade off between different symptoms and risks when considering treatment options, and preferences for treatment characteristics and the trade-offs that individuals are willing to accept between treatment efficacy and the risk of adverse events.

The initial section of the survey instrument included a Participant Information Sheet (PIS). This was followed by consent procedures and a differentiation question asking respondents to specify whether they are an individual with DLB or care partner (current or former). Logic branching was then applied to present individuals with DLB and care partners with personalized screening and demographic questions.

The subsequent section provided detailed information on the DCE attributes and related questions to assess comprehension. The six DCE attributes described in the draft survey instrument were: “risk of overall memory, thinking, and functional decline in the next 18 months,” “impact of visual hallucinations,” “impact of parkinsonism,” “impact of sleep behaviors,” “impact of fluctuations,” and “risk of brain-related side effects in the next 18 months.” The first five attributes are related to the four core diagnostic symptoms of DLB together with dementia (McKeith et al., 2017). The final attribute concerns the risk of amyloid-related imaging abnormalities (ARIA). ARIA are a reported side effect of anti-amyloid therapies in clinical trials for patients with Alzheimer's disease (AD) (Sperling et al., 2011; Filippi et al., 2022; Jeong et al., 2022). ARIA are commonly transient and clinically asymptomatic; however, ARIA can lead to exacerbation or emergence of symptoms. Severe manifestations of ARIA, including seizures, stroke and meningitis have been documented (Salloway et al., 2022; Atwood and Perry, 2023; Sims et al., 2023; van Dyck et al., 2023). ARIA of this severe nature have the potential to be reversed; however, they may require hospitalization and can be fatal (VandeVrede et al., 2020; Filippi et al., 2022; Reish et al., 2023; Solopova et al., 2023). Given that DLB pathology commonly co-occurs with AD pathology (Irwin and Hurtig, 2018), it is expected that anti-amyloid therapies will be trialed in DLB populations. The final attribute was therefore included to understand the risk tolerance of people affected by DLB.

While including all relevant possible attributes in a DCE is ideal, it can increase survey complexity and participant burden. To balance comprehensiveness and feasibility, we therefore selected a subset of possible attributes, ensuring that those central to the research question and decision context were included. Guided by our research question, evidence synthesis and consultation with clinical experts, we focused on the four core diagnostic symptoms of DLB, along with global cognitive and functional decline, due to their clinical significance. Together with a final attribute related to the risk of ARIA, this resulted in six final attributes, aligning with current practices in health-related DCEs (Soekhai et al., 2019a). However, with consideration of the capacity and vulnerability of our target population, we made additional considerations by restricting the number of levels and using color-coding which has been reported to reduce DCE choice task complexity (Jonker et al., 2019). Since DCEs and BBS are novel for this population, we are also interested in the tolerability of these methods.

**Figure 1:**
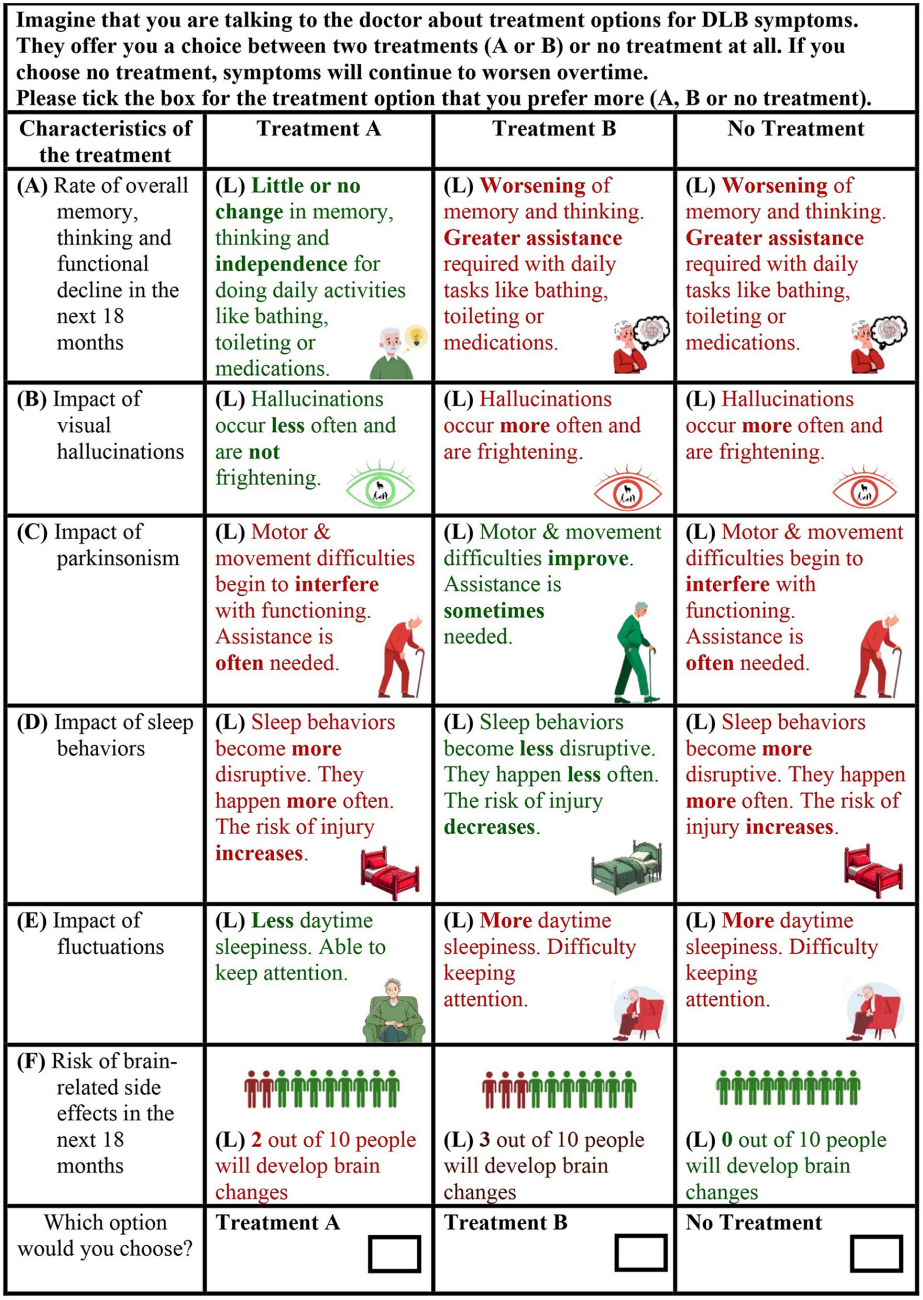
Example discrete choice experiment choice task from the draft survey instrument. The three alternatives were described by six attributes (labeled “A–F” in the example). The attributes varied across different levels (labeled “L” in the example).

After the description of three attributes, a practice DCE task with a reduced number of attributes was introduced. Subsequently, descriptions of the remaining attributes were provided, followed by eight DCE choice tasks (Figure 1). Each DCE choice task featured three hypothetical treatment alternatives. The alternatives were described by the six attributes which varied across different levels. Across the choice tasks, the attribute levels describing two of the alternatives (treatment A and treatment B) varied, while the attribute levels describing the third alternative (no treatment) remained fixed. The “no treatment” alternative acted as an opt-out.

**Figure 2:**
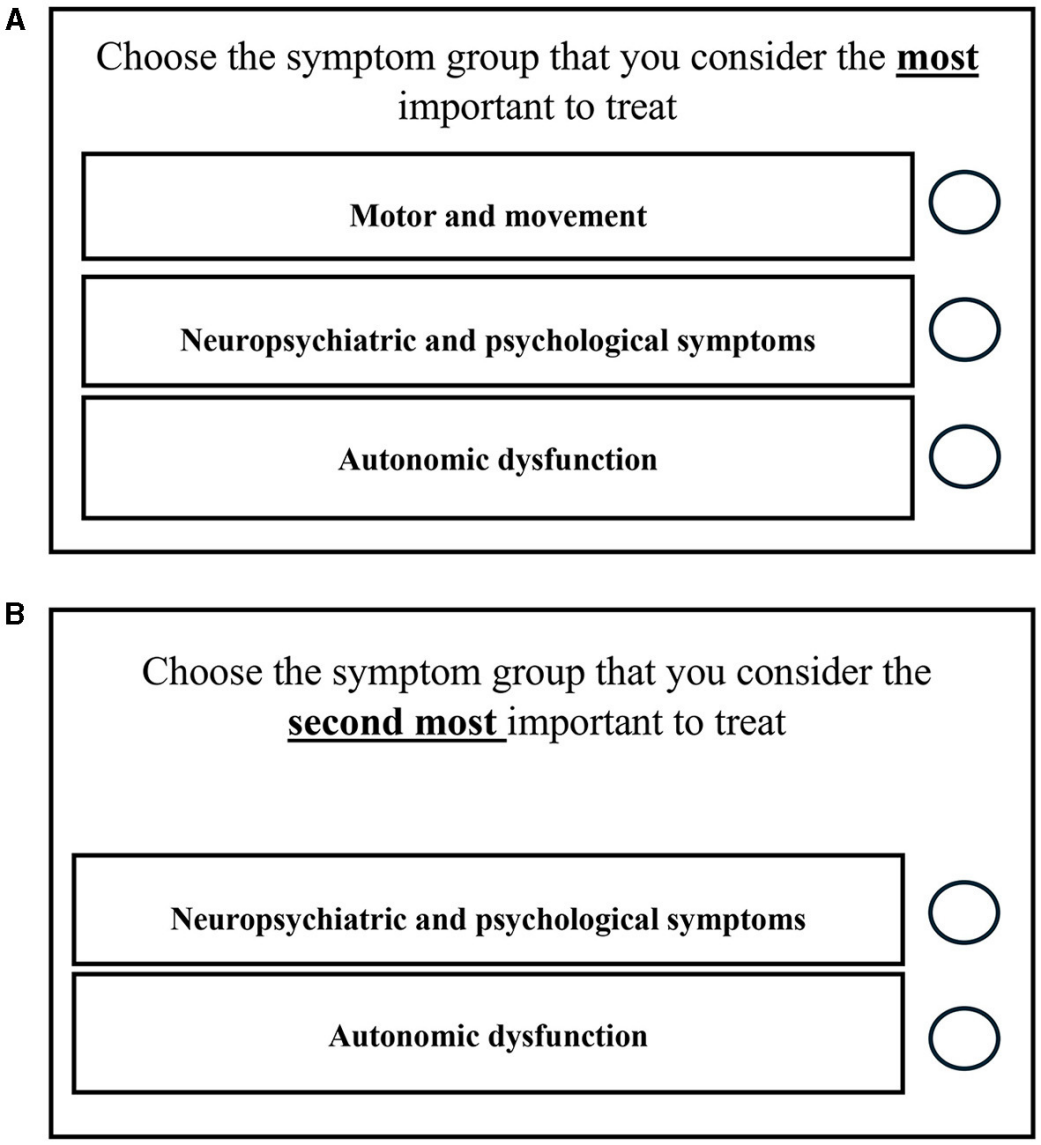
Example best-best scaling choice task from the draft survey instrument. Once the participant selects the most important symptom group (item) to treat in the first part of the task **(A)**, this item will disappear. The participant is then asked to choose the most important symptom group to treat from the remaining two items **(B)**. In the example provided, one option has been removed to show what it would look like if the participant selected “motor and movement” in the first part of the task.

The next section began with an overview of the six items included in the BBS: “motor and movement difficulties,” “memory and thinking,” “autonomic dysfunction,” “neuropsychiatric and psychological symptoms,” “sleep-related concerns,” and “fluctuating cognition.” Following this description, six BBS choice tasks were presented (Figure 2). Each BBS choice task displays a subset of three items from the full set of six items. Respondents always select from a choice of three items in each BBS choice task. The items in each task vary across the choice tasks, providing the analyst with a relative ranking of all the items. In each task, respondents were first asked to select their most preferred (i.e., best) symptom group to prioritize for treatment. Next, respondents were asked to choose their most preferred symptom group to prioritize for treatment from the remaining two options (i.e., second-best). Following both choice experiments, respondents viewed a series of questions about their preferences for treatment characteristics and their tolerance of fatal risks resulting from adverse events associated with a hypothetical treatment.

### 2.3 Involvement event

Involvement of the LBD RAG at this stage of the study was conducted as a half-day event in September 2023, held in person on university premises. An alternative approach to the instrument development process was employed whereby end-user engagement and pretesting were carried out simultaneously during the involvement event. A co-design approach was adopted with RAG contributors actively providing input to refine the survey. A hybrid approach utilizing a focus group discussion with breakout interviews was employed to capture substantial input from contributors within a resource-limited context. The procedures for the focus group and interviews are detailed below. As the purpose was to inform the evolution of the survey instrument rather than to collect qualitative data, neither the focus group discussion nor the interviews were audio-recorded nor transcribed verbatim.

As recommended during pretesting, peer-review by other scientists was also conducted (Johnston et al., 2017). This occurred following LBD RAG input. Two internal peer-reviewers, selected for their relevant specialist interests, their clinical experience with our target population and the absence of identified conflicts of interest, independently reviewed the study documentation and provided feedback from a methodological perspective on the survey instrument's ability to address the research aims.

#### 2.3.1 Focus group procedure

The focus group, facilitated by three members of the research team, lasted ~150 min, with breaks incorporated and refreshments provided. All RAG contributors participated in the discussion as a single group. During the discussion, one researcher (observer) was assigned specifically to take notes, and two research nurses offered practical support to RAG contributors. Before the focus group commenced, all RAG contributors were briefed on the study's aims and objectives as well as the importance of PPI in the research. This was followed by an introduction to DCEs and BBS. The focus group discussion was semi-structured, and RAG contributors were given paper copies of the study documentation, supplemented by a PowerPoint presentation.

First, each DCE attribute description was reviewed in turn to seek advice on comprehensibility and accuracy based on the lived experiences of the RAG contributors. The RAG contributors were also asked to provide feedback on the appropriateness of the graphics used to represent each attribute (shown in Figure 1). The researchers developed the graphics by utilizing a blend of freely available graphics sourced online and Microsoft's Image Creator, an image generation tool. After reading each attribute description, contributors were invited to respond to questions such as, “What do you think we are communicating” or “Is there anything missing in the description or that is inaccurate?” When feedback was provided by someone, the other contributors were asked if they agreed. If there was disagreement, the group collaborated to suggest improvements.

The discussion of the DCE attribute descriptions was followed by a review of key study documentation including the PIS, screening and demographic questions and BBS attribute descriptions and tasks. Finally, RAG contributors discussed the end-of-survey questions regarding important treatment characteristics and risk tolerance for fatal adverse events.

#### 2.3.2 Face-to-face interview procedure

Following the initial whole group discussion of the DCE attribute descriptions involving all RAG members, breakout face-to-face interviews commenced in an adjacent room. These interviews ran parallel to the ongoing focus group. RAG contributors, either as patient-care partner dyads or individually, sequentially withdrew from the focus group to complete the interviews. Two researchers, who also left the focus group, facilitated these interviews, leaving one researcher to continue leading the focus group discussion. Upon completing their interview, the contributors rejoined the ongoing focus group discussion.

The interviews were conducted to pretest example DCE choice tasks. The aim was to assess the feasibility of the tasks and collect feedback from the RAG contributors on the accessibility and acceptability of the choice tasks. Printed copies of six choice tasks were provided, and contributors completed them in the presence of two researchers, one of whom took notes. The order of the choice tasks was designed to progressively increase in complexity to determine the point at which respondents experienced fatigue or resorted to the use of simplifying heuristics i.e., decision-making strategies which allow individuals to make choices with less cognitive effort, such as choosing to ignore some attributes (Veldwijk et al., 2023).

To capture feedback, observations were made regarding contributors' reactions to the information presented, such as signs of confusion or hesitation. In addition, think-aloud and concurrent and retrospective probes were used. This included questions such as, “How did you reach that answer?” and “Did you find that easy or difficult to answer?” Think-aloud feedback allowed the researcher to assess choice validity by evaluating whether contributors practiced compensatory decision-making (trading attributes against each other), or whether they used simplifying heuristics which would impose challenges for modeling. If think-aloud data was not provided, a researcher probed contributors on their decision process. During the interviews, RAG contributors also highlighted challenging or confusing aspects of the choice tasks. This led to discussions between the two researchers present during the interview and the contributors on possible amendments to clarify areas of confusion.

After completing five-six DCE choice tasks, RAG contributors were asked about the difficulty of the tasks, the appropriateness of the hypothetical scenario, and whether they perceived eight choice tasks to be manageable.

### 2.4 Feedback and improvements

Although one researcher was assigned to note-taking, all three researchers made notes throughout the event. After the session, the three research team members collated the written information they had collected, and notes were cross-referenced for triangulation. Considering that only one researcher was facilitating the focus group at one point, and therefore only their notes were available for that part of the discussion, we conducted member checking with the RAG contributor who is included as a co-author on the paper (EW). This contributor reviewed the paper to verify the accuracy of the reported outcomes.

The recommendations arising from RAG input were then categorized *post-hoc*. The day following the involvement event, the study coordinator, who was involved in the event, contacted contributors to express gratitude for their participation and inquired if they had any feedback on the PPI experience. These phone calls were conversational in nature and not recorded for the purpose of data collection; rather, informal feedback served to provide guidance for the research team on future PPI activities in the study.

## 3 Results

**Figure 3:**
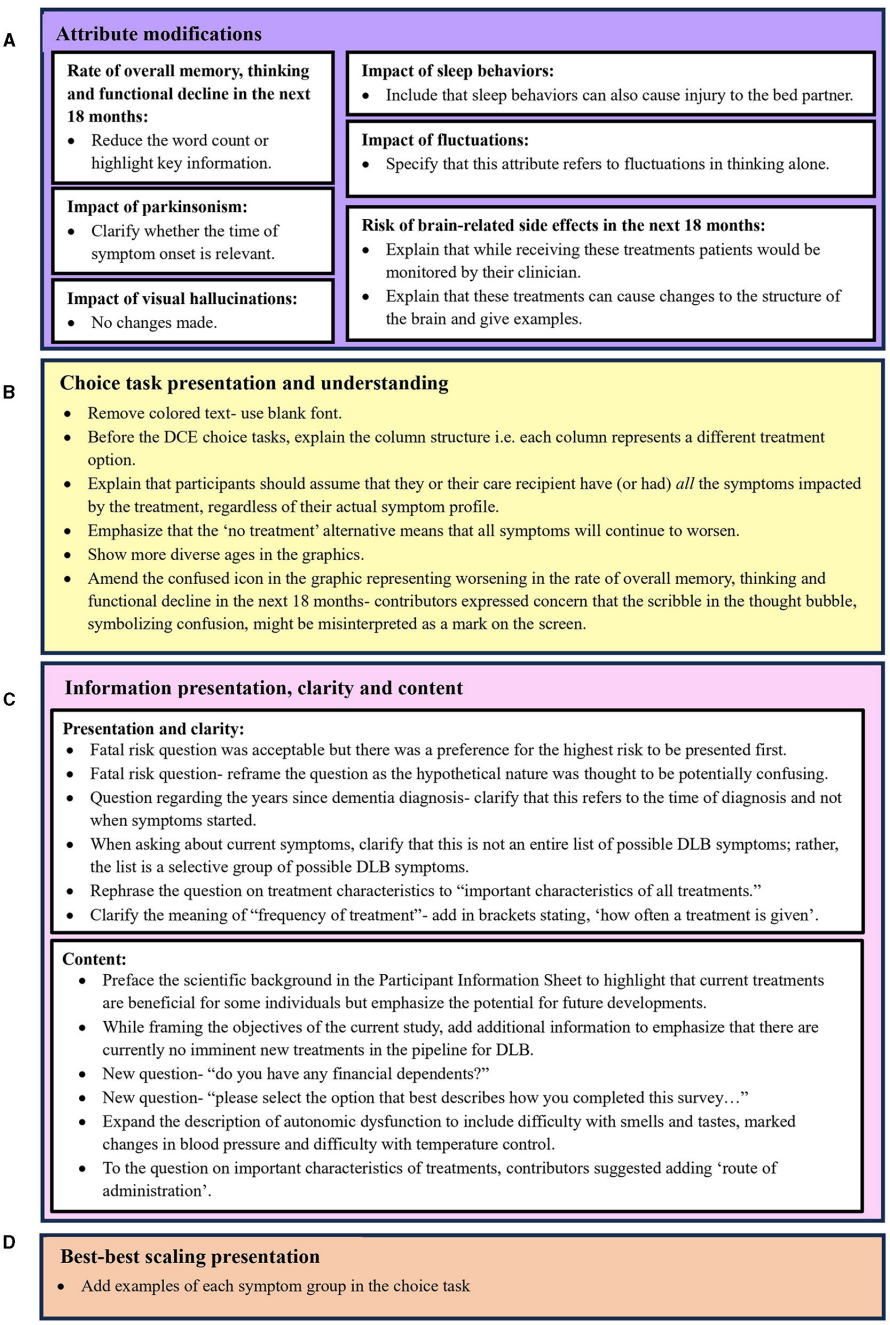
Improvements to survey instrument arising from contributor feedback across four categories: **(A)** Attribute modifications, **(B)** Choice task presentation and understanding, **(C)** Information presentation, clarity and content, and **(D)** Best-best scaling presentation.

Overall, RAG contributors provided positive feedback about the proposed research, and peer-reviewers were satisfied from a methodological perspective. In total, one focus group, two individual interviews and four dyadic interviews were conducted. The feedback provided by the RAG was considered by the research team, and changes to the SP methods and survey instrument were made as appropriate. The full list of changes that arose based on feedback from the RAG is displayed in Figure 3 according to their respective category.

### 3.1 Improvements to the survey design

RAG contributors valued the PIS. Although they found it lengthy, they acknowledged the necessity of the information. However, RAG contributors requested that the “purpose of the study” section of the PIS highlight that while there is currently an absence of treatments that alter the disease course in DLB, the study aims to inform the design of future studies for new treatments. This additional information was incorporated.

With regards to the feasibility of the DCE, RAG contributors felt that, for individuals with mild DLB, eight choice tasks would be manageable if there was the option to pause the survey and when care partner support was available. Figure 3A illustrates the amendments aimed at improving respondents' understanding of the DCE attributes. All attribute descriptions underwent revisions, except for the “impact of visual hallucinations” attribute. An important change was made to the “risk of brain-related side effects in the next 18 months” attribute. Consultation was sought from the RAG to address this attribute with sensitivity and clarity. Initially described as “brain changes,” some RAG contributors expressed concerns about potential misinterpretations of clinically beneficial brain changes. Consequently, clarifications were made indicating that treatments could lead to adverse or unintended changes to the brain, such as edema and/or stroke. These examples were informed by the manifestations of ARIA in clinical trials investigating monoclonal antibodies for AD (VandeVrede et al., 2020; Filippi et al., 2022; Reish et al., 2023; Sims et al., 2023). Given ethical considerations regarding discussing serious adverse events (SAE), the research team also consulted the RAG for their perspectives on the appropriateness of this attribute and the examples of SAE. Contributors were asked whether discussions of adverse events caused distress, and although varying levels of comfort were noted, there was a consensus on the importance of acknowledging potential adverse events because it is an important factor influencing treatment preferences. To mitigate potential distress for prospective participants, RAG contributors suggested including additional contextual information within the attribute description describing how these risks would be managed. Therefore, in the attribute description we clarified that individuals receiving treatment with a risk of SAE would be closely monitored by their clinician. This was based on the monitoring practices employed in phase 2 and 3 clinical trials to detect and manage ARIA in AD patients (Cummings et al., 2022, 2023).

Figure 3B lists the modifications aimed at improving the presentation and understanding of DCE choice tasks. Given that people with DLB may experience visuoperceptual difficulties, there was consensus that the colored text used for the attribute levels was difficult to read (Figure 1). Following RAG advice, the colored font was switched to black text for improved legibility, while retaining color-coding in the supporting graphics. Contributors did not express any concerns regarding font size or style. RAG contributors also highlighted the potential difficulty for individuals with dementia to interpret the information in the DCE when it is presented in columns. Given that the use of a matrix in DCEs necessitates a columnar presentation, the research team worked with RAG contributors to develop clear instructions on how to read the choice task.

During the pretest interviews, confusion arose among some RAG contributors regarding whether they should disregard attributes (symptoms) that are currently not relevant to them or their loved one. Changes to improve the understanding of the choice question therefore included improving instructional clarity. RAG contributors were asked if they felt it was possible to imagine having all the symptoms before making a choice, which they felt was feasible. This addition aimed to address possible attribute non-attendance and any misinterpretations that the appearance of worsening symptoms in the choice tasks, currently not experienced by them or their loved one, was indicative of developing new symptoms.

However, although RAG members could make choices as though they were experiencing all the symptoms, the researcher conducting the interview observed that certain members expressed apprehension regarding symptoms not presently affecting them. Consequently, they often opted for the “no treatment” alternative. Although selecting the opt-out provides valuable insights for the analyst, it may diminish statistical power as attribute level information is not collected from every respondent. Therefore, a significant adjustment resulting from the pretesting phase was the inclusion of a forced-choice question after a respondent chose “no treatment”, prompting them to indicate their preference if only treatment A or B were available.

Figure 3C lists the changes made to meet RAG contributors' preferences for how information was presented, to improve the clarity of the information presented, and to add additional content suggested by RAG contributors. In particular, RAG contributors were consulted about whether they felt that the consideration of fatal risks associated with treatments could be distressing for potential participants. All RAG contributors felt that this was not distressing; however, they preferred that the highest risk be presented first. Input from the RAG also prompted the inclusion of new content in the survey (Figure 3C). This included the addition of a question concerning financial dependents, identified during focus group discussions as a potential influence of treatment preferences. Also, it was unanimously recognized that support from care partners may be required for individuals with DLB to complete the survey. This led to the inclusion of a question that captured the extent of support provided by care partners to individuals with DLB when completing the survey. In addition, RAG contributors referred to the importance of the treatment administration route as an important determinant of treatment acceptance. This led to its inclusion in a question on important treatment characteristics.

The RAG felt that the BBS tasks were accessible and would not burden participants. However, as illustrated in Figure 3D, contributors recommended including example symptoms related to each symptom domain within the choice task.

### 3.2 Feedback on the personal and public involvement experience

**Figure 4:**
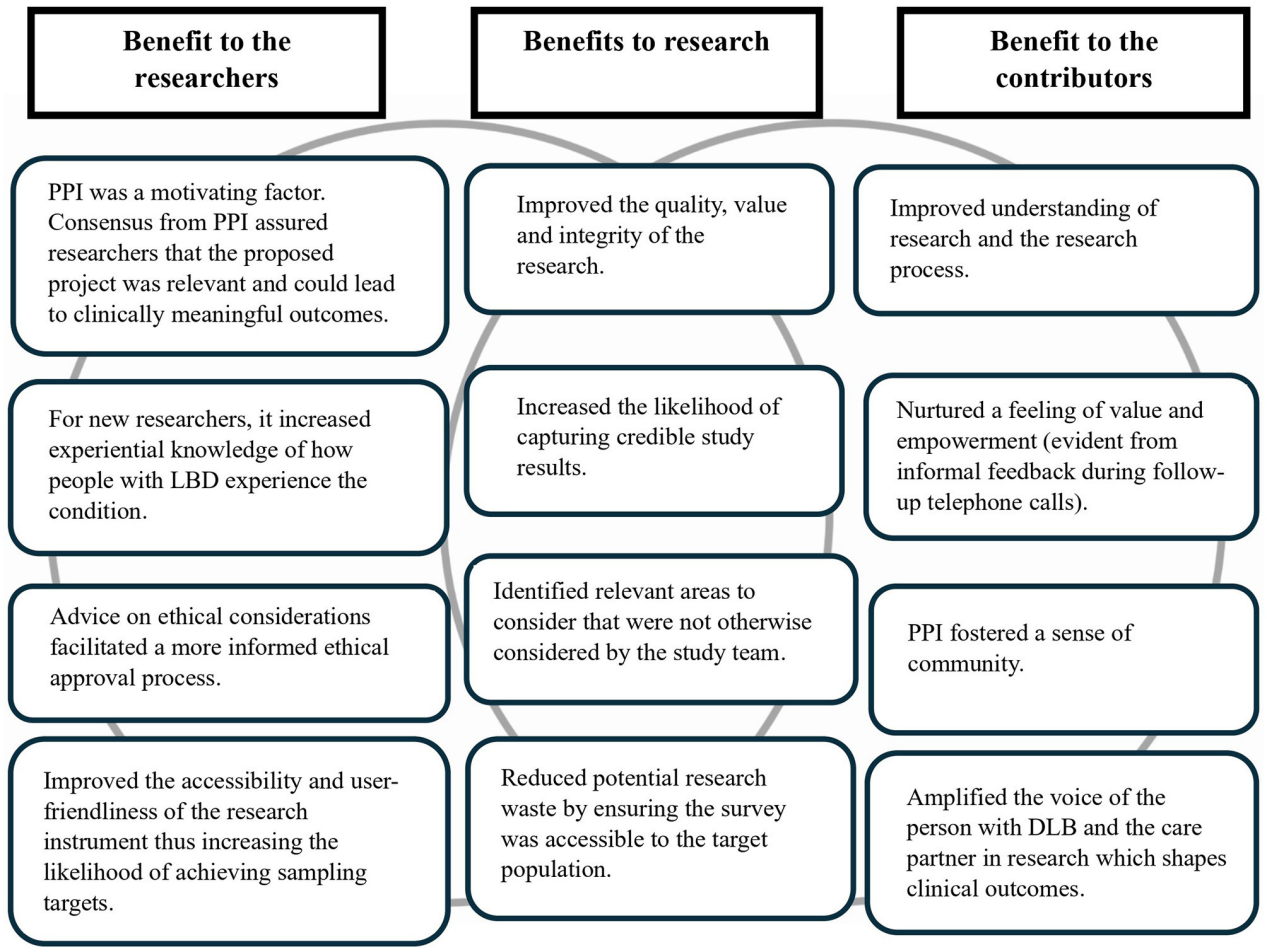
Mutual benefits of the personal and public involvement activity.

Feedback obtained from RAG contributors on their experience of being involved during the design of the study was overall positive. Feedback during the telephone calls and discussions among the research team revealed a mutually beneficial relationship arising from the PPI process (Figure 4). However, some challenges were expressed by RAG contributors including difficulty for care partners to express their opinions in the presence of care recipients, and feelings of fatigue among some members with LBD. Despite these challenges, all RAG contributors expressed an interest in continuing to act as study contributors.

## 4 Discussion

In this study, an alternative approach to survey instrument development was employed to gain valuable insights tailored to individuals with DLB and their care partners. The alternative survey development approach, which combined recommended steps for end-user engagement and pretesting (Janssen et al., 2016; Campoamor et al., 2024), was necessitated by resource constraints and the vulnerable nature of the population. The approach is detailed alongside the specific outcomes resulting from involvement.

Inclusion of patient and public partners in the development of preference elicitation methods is increasingly acknowledged as a valuable mechanism for informing methodological choices and improving the relevance of preference research (Aguiar et al., 2021; Shields et al., 2021). However, PPI is rarely reported in preference studies despite often being mandated by funders (Shields et al., 2021). By providing a comprehensive report on the development process of our survey instrument, we not only foster transparency but also acknowledge the substantial value of PPI input in preference research.

The co-design process undertaken during survey development resulted in tangible modifications to the survey instrument. For example, we encountered unexpected challenges with the color-coding of the DCE text among RAG contributors experiencing visuoperceptual difficulties due to DLB. This contrasted prior findings suggesting potential benefits of color-coding for reducing DCE task complexity (Jonker et al., 2019). While contributors demonstrated a good understanding of the DCE attributes and BBS symptom domains, understanding the DCE attribute related to brain-related side effects posed some difficulty, prompting collaboration to refine the attribute description. Additionally, RAG input proved invaluable in reducing the risk of potential participant distress associated with this attribute. Ensuring appropriate communication of this risk attribute was crucial. Experiential knowledge and perspectives from RAG contributors played a critical role in achieving this. Collaborating with contributors, the decision was made to include an explanation that close monitoring would be carried out by clinicians during treatment, as is stated in the appropriate use recommendations for emerging monoclonal antibodies for AD (Cummings et al., 2022, 2023).

The involvement of RAG contributors went beyond refining the survey. RAG contributors also generated additional ideas, reflecting an actively engaged, co-design approach that significantly contributed to the relevance of the survey instrument. By involving those directly impacted by DLB, we ensured that the survey captured essential perspectives and was person-centered, a quality indicator of stated preference methods (Janssen et al., 2017). While this led to direct benefits to the research, benefits for both the research team and contributors were also noted (Figure 4). These echoed previous reports of the mutual benefits of PPI in research (Aries et al., 2021).

The effective partnership may have been facilitated by efforts made to minimize power imbalances, an inherent issue in PPI (O'Shea et al., 2019). This included offering remuneration for contributors' time and reimbursement for their expenses. We also implemented recommendations whenever possible and reached compromises when necessary. Adaptations were also made to support contributors with cognitive impairment, including the provision of communication cards and frequent breaks. Since individuals affected by DLB may encounter challenges with speech fluency (Ash et al., 2012), the communication support cards (which read “I would like to speak”) served as non-verbal cues that contributors could display during discussion to express their desire to contribute. However, none of the contributors utilized the communication support cards on this occasion. Nevertheless, these efforts likely contributed to fostering a positive environment that encouraged contributors to share their views.

Inclusive opportunity is a key standard outlined in the UK standards for public involvement (Crowe et al., 2020). Rather than intensive pretesting across multiple waves which could burden individuals with cognitive impairment, involvement across a single half-day event enabled inclusive opportunity for people at both mild and moderate stages of dementia. This avoided the need to rely on a homogenous sample of individuals at the early stage of dementia. Training for PPI contributors is also encouraged in the UK standards (Crowe et al., 2020). Challenges with conducting PPI in the design of preference elicitation surveys has been related to the appropriate level of training provided to contributors (Al-Janabi et al., 2021; Shields et al., 2021). In the current study, RAG contributors were introduced to the concept of DCEs and BBS, but they were not provided with structured training on these methods. It is therefore possible that this limited the input from contributors. Nevertheless, it is essential to discuss training expectations with contributors, especially in vulnerable populations.

We opted for informal, unstructured conversations to evaluate the impact of PPI input at this stage of the study because we believed it would be the least burdensome approach for contributors. However, use of existing tools such as a public involvement log or the Public Involvement Impact Assessment Framework could have enhanced the evaluative process (The PiiAF Study Group, 2014). Further discussion of the challenges and reflections on PPI in the study are reported using the GRIPP2-SF (Table 1).

The objective of this paper is not to assess the strengths and limitations of focus groups and interviews as research methods or to evaluate the integration of these methods for data collection. Instead, the focus is on evaluating the value of the alternative survey development approach within a resource-limited context. The reported benefit of focus groups as a flexible, efficient method for discussing concepts and language was evident in this study (Johnston et al., 2017). Additionally, the heterogeneity of perspectives in the focus group enhanced the richness of feedback received and fostered a sense of community among PPI contributors which was observable in their interactions. While focus groups have been criticized for their lack of ability to facilitate individual exploration and “groupthink” (Busetto et al., 2020), we were able to offset this through utilizing simultaneous breakout interviews. The breakout interviews allowed for independence of individuals responses and reduced the time between discussion and recall for people with cognitive impairment.

### 4.1 Limitations and future directions

The traditional pretesting process of implementing iterative changes across waves was not feasible using the current approach. While iterative modifications are advantageous because they allow for revisions to be assessed, it was felt that, in the current context, extensive pretesting would impose burden on contributors and compromise the heterogeneity of the RAG by forcing reliance on people at earlier stages of dementia. Future studies may explore the benefits of two sequential focus groups with iterative changes made following session one and reviewed at session two. However, the potential benefit of employing this approach, in contrast to the methodology adopted in this study, should be weighed against the associated risks of burden and should take account of the constraints of PPI budgeting.

Future studies may also consider individual interviews for care partners and people with DLB to potentially capture richer responses. However, the dyadic nature of the interviews reflects real-life clinical situations where the patient's cognitive, behavioral and functional capacities are often discussed with patient and care partner dyads. If individual interviews are considered, investigators should work with contributors to determine their preferences and to avoid the risk of causing unnecessary stress or anxiety for individuals with DLB.

Moreover, sociodemographic and clinical data were not collected on LBD RAG contributors. However, the LBD RAG is a local RAG comprising individuals recruited exclusively from Northern Ireland and so the opinions of individuals with DLB and their care partners from other geographical locations were not adequately represented. Similarly, all RAG contributors with DLB resided at home, thus excluding the perspectives of individuals with DLB in care settings, who may have more advanced dementia. Academic researchers at the ECRED have highlighted the ethical challenges associated with including individuals from care settings, particularly in circumstances lacking resources such as transportation to facilitate their participation (Warran et al., 2023). Future studies could consider using video conferencing platforms or arranging transport to facilitate the participation of those residing in care settings to participate in PPI initiatives. DLB is also a highly heterogeneous disease (McKeith et al., 2017) and, as with all involvement work, the views of the RAG may not reflect the views of all individuals with DLB and their care partners.

We also acknowledge that the fatigue experienced by some contributors could have limited their engagement. To address this, future studies could consider conducting interviews and the focus group across 2 days or offering fatigued individuals the option to complete interviews on a subsequent day, either in person or virtually. This approach may also be supportive given that people with DLB can experience fluctuating cognition.

Finally, while it is felt that the outcomes reported informed a survey instrument capable of more accurate data collection, there is no evidence to support a connection between the implemented modifications and the quality of the resulting data.

## 5 Conclusion

This work contributes to the emerging literature on pretesting in SP surveys and the value of PPI in SP research. Involvement of the PPI RAG resulted in a more valid and reliable survey design that better addressed the needs and preferences of individuals with DLB and their care partners. In a resource-limited context, the approach taken was a feasible and pragmatic mechanism for improving the survey design through feedback from the target population. As recognition of the value of SP methods to inform regulatory decision-making continues to increase, their use in DLB and other dementia populations is expected to increase. Future studies should further explore collaborative survey development approaches with this population, with authors encouraged to share their strategies and outcomes to inform best practice.

## Data availability statement

The original contributions presented in the study are included in the article/supplementary material, further inquiries can be directed to the corresponding author.

## Ethics statement

Ethical review and approval was not required for the study on human participants in accordance with the local legislation and institutional requirements. Written informed consent from the patients/participants was not required to participate in this study in accordance with the national legislation and the institutional requirements.

## Author contributions

PD: Conceptualization, Data curation, Formal analysis, Funding acquisition, Investigation, Methodology, Project administration, Resources, Visualization, Writing – original draft, Writing – review & editing. AS: Writing – review & editing, Investigation. EW: Writing – review & editing. AP: Conceptualization, Supervision, Writing – review & editing. NM: Conceptualization, Supervision, Writing – review & editing. MB: Conceptualization, Methodology, Resources, Supervision, Validation, Writing – review & editing. JK: Conceptualization, Funding acquisition, Investigation, Methodology, Resources, Supervision, Validation, Writing – review & editing.
